# A System of Human Anatomy, from the French of M. Cloquet

**Published:** 1829-07-01

**Authors:** 


A SYSTEM OF HUMAN ANATOMY. TRANSLATED FROM THE FRENCH OF H. CLOQUET, M.D. By *Robert Knox*, M.D. F.R.S. E. &c. With Notes and a corrected Nomenclature. 1 vol. 8vo. pp. 840. Edinburgh, 1828.

ELEMENTARY works are pouring in upon us from every quarter. In one number of our Journal we are called upon to notice two important publications on anatomy—one by Mr. Quain, a compilation—the present by Dr. Knox, a translation. These two works are very differently constructed—the former on the English model—the latter on the French. The distinctive characters of the two plans will be best understood by the following quotation from Dr. Knox's preface.

"The peculiar excellence and merit of British anatomical writers seems to be mainly connected with the originality of the views brought forward in their monographs, and even in their more voluminous systematic works: the extreme accuracy of detail, whenever the subject to be described leads to practical results; the splendid and happy physiological and pathological deductions which abound in their writings; but above all, with the discovery, if I may say so, and perfection of those descriptions of the more important surgical regions of the body, a work begun and finished solely by British anatomists and surgeons.

"These seem to me (for I must speak with diffidence, aware of my inherent nationality,) a few of the excellencies which abound in British writers; by their very presence they exclude other good qualities, which, though of a more humble and inferior cast, are yet of the utmost consequence to the anatomical student.

"The excellence here alluded to, which we in vain look for in British, and but seldom fail to meet with in Continental writers (so religiously have they copied each other from Winslow to the present day,) is that of presenting to the anatomical student a clear, methodical, and concise, yet minute description of all the parts of the human body, with brief allusions only to the uses of the parts, and with none of those alarming digressions, interlarding and disfiguring the works of English anatomists." iv.

That the plan of intermixing pathology, surgery, and physiology, with pure anatomy, is the prevailing taste of the English, there can be no doubt; and that it has its attractions and advantages it would be useless to deny. Yet, on the principle of division of labour, and utility of *method*, it would not be difficult to shew that terse and minute anatomical description, uninterrupted by surgical or physiological digressions, is a species of knowledge which is of great utility in the tuition, and consequently in the learning of anatomy. It is curious that the English have adopted the diffuse but original style of Galen, Vesalius, and some other of the older anatomical writers—while the French have servilely copied the rigid and exact style of Winslow.

"And yet with all the contempt one naturally feels for mere copyists, it must be admitted that the model followed by the Continental anatomist is, in many respects, better than our own, and that the diffuse wandering style of Galen and Vesalius, with their monstrously absurd theories and endless repetitions, will not stand a comparison with the concise and energetic manner of Winslow, his mechanical accuracy, and his brief yet perspicuous manner." iv.

The followers of Winslow, on the Continent, among whom may be enumerated Soëmmering, Sabatier, Boyer, Portal, Bichat, Cloquet and others, have founded their claims to merit on the fidelity of their descriptions, as *text-books*, aiming at no higher character—" and if," says Dr. Knox, " we deny to them the smallest title to genius or originality, the praise of clearness of description, admirable general perspicuity, happy arrangement and minute description, cannot possibly be withheld."

After the peace of 1814, and when a free communication was established between this country and France, surgical anatomy was imported into the latter country—became quite the rage—and appears to have been carried to an almost ridiculous excess. The following extract from Dr. Knox's preface will excite a smile, while it shews that, in the " Intellectual City," there are feuds and jealousies going on, as well as in the " Modern Babylon."

" They dispute about the *discovery* of parts which all anatomists have seen since the days of Herophilus; but such is the method and regularity of their works, and so laudably anxious are they that no important or novel consideration should escape them, that Mr. Blandin, (I must choose one specimen), describing all the possible surgical anatomy of, and all the surgical operations which can possibly be performed in and about the head, (which he invariably designates by the imposing philosophic name of ' Encephalic extremity of the Trunk'), very gravely, and with the best faith in the world, informs his readers, that ' the head cannot be removed by amputation in the living subject without stopping respiration, and producing other inconveniences which render this operation unhappily inadmissible."* iv.

Dr. Knox passes some other sly criticisms on his countrymen. He observes that the Continental anatomical writers describe the anatomy of the whole body with equal care, and with nearly equal minuteness. They do not presume to prejudge the relative importance of certain regions of the body, and to measure the degree of attention which the student and surgeon should devote to some, and the measure of neglect to other parts of the body.

" They do not inculcate to their readers that the muscles of the back are altogether unimportant; a minute knowledge of the bones unnecessary; the smaller arteries not worth remembering, and the anatomy of the nerves absolutely useless. These doctrines, wherever or by whomsoever taught, are peculiarly dangerous to the student; if listened to for a moment, they must destroy his views for ever.—*Pref.* vii.

The following sarcastic remarks are, we conceive, levelled at some promi-

---

* " ' Mr. Blandin ought not to despair: we now know, by accurate experiments made before respectable witnesses, that the human body may be ripped up alive, and yet the person escape, nay even recover. Limbs are hewn off *against time*; if one man amputates at the hip-joint, and requires only five minutes for his operation, another boasts of his greater expertness in quartering the body, as he requires only a minute and a half to perform a similar operation; and although the operation should accidentally be unnecessary, and therefore cruel and scandalous, and moreover fatal, he advertises it in the daily newspapers, ' that his light may not shine under a bushel.' It is said, moreover, that an attempt is to be made to remove the upper jaw in a case of diseased face; now, should the cranium of the patient happen to be furnished after the same fashion as the operator, its removal may possibly be unattended by any bad results. The case may even figure as one of ' amputation of the head.' "

nent characters in Edinburgh, whose names we shall not attempt to decipher, though it might not be extremely difficult to unriddle the allusion.

"There are *many* persons, indeed, some of them, I regret to say, teachers of anatomy, who seem to think that anatomy in itself is the most tiresome, disgusting, and uninteresting of all studies; and, proceeding on this notion, pregnant with dangerous consequences to the profession and to the science, have taught anatomy as a mere appendage to surgery; and carrying into a liberal profession their own empirical views, have rejected, and anxiously taught others to reject every anatomical fact which did not lead to the 'dextrous performance of some cunning and skilful operation in surgery, the execution of which should be most profitable to the surgeon;' or they have compounded it, to use a professional phrase and metaphor, with medicine, physiology, and pathology, as if anatomy were a nauseous draught, requiring to be masked by some palatable, overpowering ingredient. And this is the language held of a science, most exact in itself, most interesting and important to the human race, the foundation of the philosophy of living beings, and the only basis on which the superstructure of medicine, surgery, and physiology can be raised." vii.

We shall not attempt to decide the point between the French and English anatomists, respecting the plan of anatomical works. The English, as we said before, mingle surgery and practical remarks with descriptive anatomy—the French prefer pure anatomical description. The following is the sentiment of Dr. Knox, a teacher of anatomy, and one who ought to be a judge.

"Systematic compilations or treatises on human anatomy which simply describe the structure, appearance, and general situation of the organs to each other, constitute a species of writing seldom attempted by British writers: and yet these are of all others, the kind of works most essential to the student in anatomy; or rather, I should say, to him indispensable. Our manuals for the use of the dissecting-room, so admirable for the details of surgical anatomy, are generally vague, deficient, and not unfrequently incorrect in their descriptions of many parts of the body. Our systematic works seem avowedly written as much for amusement as for instruction, so numerous and extraordinary are the digressions they contain. But whether or not these criticisms be strictly just, I felt I could not possibly dismiss from my mind that important truth, that works conceived and executed after the model of Winslow, Sabatier, Portal, Soëmmering and others, do not exist in the English language; a truth daily obtruded on me by my duties as a teacher of anatomy, and unhappily annually giving rise to the statement required to be made by me to one of the most numerous classes of anatomical students with which the public has ever honoured any individual, namely, that the text book of my lectures must be selected from amongst a class of authors infinitely inferior in genius, but superior in method and good taste, to those of my country." viii.

It is hardly necessary to add, that Cloquet's work has been selected by Dr. Knox as an anatomical treatise, than which nothing superior has yet been offered to the public. It is, of course, purely descriptive—and we believe the descriptions are scrupulously correct.

We can only give a single specimen of the matter and manner of this work, taken at random by opening the book nearly in the middle!

"OF THE DUODENUM.

"*a.* CONFORMATION AND GENERAL DISPOSITION.

"2095. The *Duodenum*,* by which the intestines properly so called com-

"* It is so named on account of its length being commonly estimated at twelve finger breadths."

mence, immediately succeeds to the stomach. It is less voluminous than that organ, but has a greater diameter than the rest of the digestive canal, and is susceptible of great dilatation. It occupies the deep middle part of the abdomen, where it is concealed by the transverse mesocolon or by the stomach.

"2096. The direction of the duodenum is such that it may be divided into three portions. The first, which is about two inches long, commences at the valve of the pylorus (2091), proceeds horizontally backwards and to the right, and ends near the neck of the gall-bladder, uniting angularly with the second, which has a variable length, and which descends vertically and a little to the left, as far as the third lumbar vertebra. The third portion is directly continuous with the second, with which it does not form an angle. It proceeds transversely to the left, before the vertebral column, and ends with being directed upwards and forwards, toward the upper extremity of the mesentery, above the superior mesenteric vessels, which cross its direction, and which are embraced by a kind of curve which it forms for them.

"The first portion is covered, in the greater part of its extent, by the peritoneum, and is in connection with the hepato-gastric omentum. It is often tinged yellow by the transudation of the bile. The second has no other connection with the peritoneum than that of being covered by the upper lamina of the transverse mesocolon. The third is contained between the two laminæ of that fold.

"From this disposition, the duodenum forms a kind of semicircle which circumscribes the pancreas, and has its concavity to the left, and its convexity to the right. It only appears to be kept in a fixed position in its two inferior thirds.

"2097. The relations of the duodenum to the neighbouring organs are the following: *above*, it corresponds to the liver and part of the neck of the gall-bladder; *below*, it is limited by the inferior lamina of the transverse mesocolon; *anteriorly*, it is covered by the superior lamina of this fold inferiorly, and by the stomach and right extremity of the arch of the colon above; *posteriorly*, it is applied upon the anterior and right lateral parts of the vertebral column, the right kidney, the vena cava inferior, the aorta, and the right pillar of the diaphragm. By its whole inner side, it embraces the pancreas, from which it is separated below by the superior mesenteric vessels. Its outer side is immersed in the sub-peritoneal cellular tissue, between the right kidney and the ascending portion of the colon.

"2098. The *inner surface* of the duodenum is mucous like that of the stomach. There is seen upon it a multitude of circular folds, which differ extremely in their configuration, and are very close to each other. These are the *valvulæ conniventes*. They are formed by the mucous membrane alone, and their existence is constant in all states of the duodenum. They project three or four lines in its cavity. Some of them are oblique and cross each other, or run into those next them; their length is not the same in all; they never form entire circles, only representing arches which embrace the half, two-thirds or three-fourths of the intestine, whose pointed extremities advance unequally beyond each other. Their breadth varies as much as their length. The use assigned to them is that of retarding the progress of the alimentary substances for the purpose of favouring the absorption of the chyle. The reticular tissue which we pointed out at the inner surface of the stomach shews itself in the depth of the grooves by which they are separated.

"2099. There is also observed in the interior of the duodenum, at the point of union of the second and third curves, a small tubercle, at the summit of which are seen the united or isolated orifices of the choledochus and pancreatic ducts.

"2100. At its lower part the duodenum is continuous with the small intestine, without any very distinct line of demarcation being observed.

"2101. From what we have just said, it has already been seen that the duo-

"*b.* ORGANIZATION OF THE DUODENUM."

denum is not, like the stomach, invested with a serous membrane, the peritoneum being only applied upon it in a small portion of its extent. It is to this partial deficiency of the peritoneal coat that the intestine owes the faculty of dilating to such a degree as almost to equal the stomach in size.

"2102. *Muscular Membrane* or *Coat.* It is pretty thick. All its fibres are transverse or circular, and have a great similarity to those of the stomach. The layer of dense and solid cellular tissue which unites it to the mucous membrane was called the *Nervous Coat* by the older anatomists.

"2103. *Mucous Membrane* or *Coat.* It is reddish, very soft, spongy, villous, and as if downy. It is of its replication that the valvulæ conniventes are formed. It possesses all the characters of the internal membrane of the stomach, and is truly continuous with it (2089.) Between it and the preceding coat, there is observed a great quantity of flattened mucous follicles, the orifices of which are more visible than in the stomach.

"2104. The arteries of the duodenum are very numerous, and come from the superior mesenteric, pyloric, pancreatic, and gastro-epiploic arteries. Its veins exactly correspond to the arteries. Its lacteals and lymphatics go to the ganglia situated above the pancreas. Its nerves come from the solar plexus (1844.)" 609.

Whatever difference of opinion may exist as to the superiority of the French or English plans, there can be no doubt that the present work offers a more dense and accurate description of the structure of the human body than can be found in any work of similar size in the English language.

---

